# Electronic, optical and thermoelectric properties of Fe_2_ZrP compound determined *via* first-principles calculations

**DOI:** 10.1039/c9ra04736k

**Published:** 2019-08-19

**Authors:** Esmaeil Pakizeh, Jaafar Jalilian, Mahnaz Mohammadi

**Affiliations:** Faculty of Petroleum and Gas, Yasouj University Gachsaran 75813-56001 Iran e.pakizeh@yu.ac.ir esmaeil_pakizeh@yahoo.com; Department of Physics, Faculty of Science, Yasouj University Yasouj Iran; Department of Physics, Faculty of Science, Qom University of Technology Qom Iran

## Abstract

In this study, based on the density functional theory and semi-classical Boltzmann transport theory, we investigated the structural, thermoelectric, optical and phononic properties of the Fe_2_ZrP compound. The results of the electronic band structure analysis indicate that Fe_2_ZrP is an indirect band gap semiconductor in the spin-down state with the band gap of 0.48 eV. Thermoelectric properties in the temperature range of 300–800 K were calculated. Fe_2_ZrP exhibits the high Seebeck coefficient of 512 μV K^−1^ at room temperature along with the huge power factor of 19.21 × 10^11^ W m^−1^ K^−2^ s^−1^ at 800 K, suggesting Fe_2_ZrP as a potential thermoelectric material. The Seebeck coefficient decreased with an increase in temperature, and the highest value was obtained for p-type doped Fe_2_ZrP when the optimum carrier concentration was 0.22 × 10^23^ cm^−3^; the n-type doped Fe_2_ZrP had high electrical conductivity than the p-type doped Fe_2_ZrP. Thermal conductivity increased with an increase in chemical potential. Optical calculations illustrated that there was a threshold in the imaginary dielectric function for the spin-down channel. Spin-dependent optical calculations showed that the intraband contributions affected only the spin-up optical spectra due to the free-electron effects. Generally, the results confirmed that the intraband contribution had the main role in the optical spectra in the low energy infra-red and visible ranges of light. We also presented the phononic properties and found that these materials were dynamically stable.

## Abstract 2

In this study, based on the density functional theory and semi-classical Boltzmann transport theory, we investigated the structural, thermoelectric, optical and phononic properties of the Fe_2_ZrP compound.

## Graphical abstract

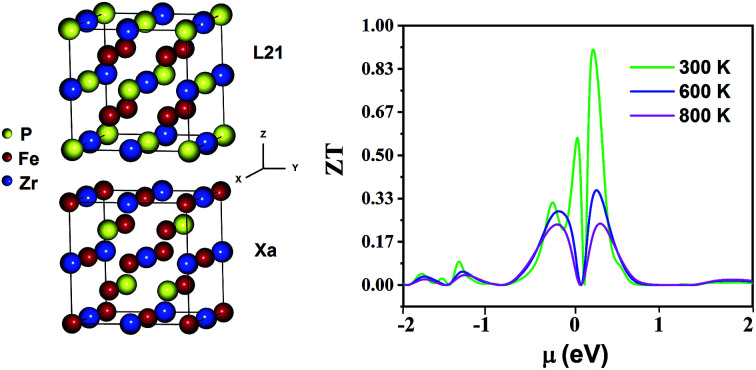

## 1. Introduction

Thermoelectric (TE) effect involves direct energy conversion by electrons in materials and is thus considered an alternative and “green” energy source. The TE effect has various advantages in industrial applications.^1–3^ The Peltier and Seebeck effects are the main TE effects. Using the Peltier effect, the TE device can cool materials. On the other hand, *via* the Seebeck effect, thermal energy can be transformed into electric energy, and this phenomenon is called TE power generation.^4^ The performance of a thermoelectric material is described by the figure of merit *ZT* = *S*^2^*σT*/*κ*, where *S* is the Seebeck coefficient, *σ* is the electrical conductivity, *κ* is the thermal conductivity, and *T* is the absolute temperature. Thus, to realize efficient energy conversion, a favorable thermoelectric material should possess high *ZT*, which indicates that a high Seebeck coefficient, high electrical conductivity, and low thermal conductivity are required for achieving efficient energy conversion.^5–8^

In recent years, Heusler compounds have been theoretically investigated, and their TE properties have attracted significant attention from researchers.^9–17^ Generally, Heusler compounds have the stoichiometric composition XYZ or X_2_YZ and crystallize in the L2_1_ structure, where X and Y are transition or rare-earth metals and Z is the main group element.^18^ These materials are half-metallic, where one spin channel shows metallicity, whereas the other spin channels are completely semiconducting. Because of this feature, half-metallic Heusler alloys can be considered as the most important class of spintronic materials.^19–23^ Half-metallic material alloys have been found in some kinds of materials such as full^16,24–29^ and half Heusler alloys,^15,30,31^ binary compounds^32–34^ and 2D materials.^35–37^

Recently, in a theoretical study, the effect of Ge substitution on the thermoelectric properties of the Heusler-type alloy Fe_2_MnSi_*x*_Ge_1−*x*_ has been investigated by Reshak.^13^ It has been reported that the Seebeck coefficient (*S*) for Fe_2_MnGe exhibits an n-type behavior over the entire concentration range. In contrast, Fe_2_MnSi has a positive S of up to 250 μV K^−1^. Comtesse *et al.*^14^ have reported the spin polarization TE properties of Co-based half-metallic Heusler compounds using the fully relativistic screened Korringa–Kohn–Rostoker theory. The transport coefficients of Co-based half-metallic Heusler materials are strongly influenced in spin polarization cases. The thermoelectric properties of the CrVNbZn Heusler compound have been investigated by Kara *et al.* based on the Boltzmann transport theory.^9^ It has been reported that a unique sharp electronic band, with highest contribution from valence electronic states, increases the TE figure of merit. Bhat *et al.* have focused on the TE performance of the ferromagnetic CoFeCrAs Heusler alloy. This material presents high *S* and huge power factor at room temperature.^11^ The thermoelectric behaviors of Ru_2_VZ (Z = Si, Ge and Sn) half-metallic full-Heusler compounds have been investigated by Yalcin.^12^ The TE parameters, such as Pauli magnetic susceptibility, electrical conductivity, *S*, thermal conductivity and power factor, were obtained by the Boltzmann transport theories; moreover, in recent years, the thermoelectric properties of Heusler compounds have been investigated *via* experimental studies.^38–41^ In this context, Chauhan *et al.* produced Zr_1−*x*_Hf_*x*_CoSb_0.9_Sn_0.1_ Heusler alloys by employing high-energy ball-milling processes and investigated the thermoelectric properties of these alloys.^38^ Their method led to the production of nanoparticles, with low thermal conductivity and high figure of merit, suitable for thermoelectric applications. The thermoelectric properties of the Zr_0.5_Hf_0.5_Co_0.4_Rh_0.6_Sb_1−*x*_Sn_*x*_ (0.15 ≤ *x* ≤ 0.5) half-Heusler alloys synthesized using a hardened steel jar and balls have been investigated by Maji *et al.*^40^ Their team found materials with a high power factor (800 μW K^−2^) and a low thermal conductivity (2.2 W m^−1^). An n-type half Heusler compound (HfZrCoSnSb) has been synthesized experimentally by Poon *et al.*^41^ They succeeded in achieving a high figure of merit (1.05) at 900 °C. This material was tested for application in p–n couple devices, and it showed good power generation efficiencies reaching 8.7% for the hot-side temperatures of about 700 °C.

A recent study based on the density functional theory and semi-classical Boltzmann transport theory was aimed at providing more detailed information about the electrical, optical, phononic and thermoelectric behaviors of the Fe_2_ZrP half-metallic ferromagnetic full-Heusler compounds. Due to the novelty of this material, only one theoretical study has been conducted on this compound by Canko *et al.*^42^ The electrical and magnetic properties of this material were studied by them. They have concluded that due to its high Curie temperature and sufficient chemical stability, this compound can be a suitable magnetic intermetallic material;^42^ moreover, although the spin-up electronic band structure is metallic, the spin-down band structure has a semiconductor behavior with the gap of 0.593 eV, and the spin-flip gap is 0.129 eV; due to this property, this compound exists in nature as well as can be synthesized experimentally. Their theoretical study indicates that the Fe_2_ZrP compound may exhibit significant promise for application in spintronic devices. To complete their study, the thermoelectric and optical properties of this material were examined for the first time in the present study.

## 2. Computational details

In this study, calculations were performed using density functional theory plane waves and pseudopotentials *via* the Quantum ESPRESSO package.^43^ The exchange–correlation term was considered by the Perdew–Burke–Ernzerhof (PBE) functional.^44^ Moreover, generalized gradient approximation (GGA) and ultrasoft pseudopotentials (US PPs) were utilized. The energy cut-off for the expansion of the wave-functions was set to 30 Ry (due to ultrasoft pseudopotentials and lattice symmetry, this energy was suitable for achieving base-state energy). The electronic wave function was expanded with the energy cutoff value of 300 Ry for charge density. Brillouin zone integration was performed over the Monkhorst–Pack^45^ 10 × 10 × 10 meshes. The lattice constant of the Fe_2_ZrP compound was optimized until the total energy converged to at least 10^−8^ Ry. Structure optimization was performed based on variable-cell (vc-relax) calculations. Considering the symmetry structure of this compound, four atoms were used in the simulation, which has been discussed in more detail in the next section. The TE properties of the Fe_2_ZrP compound were investigated with the BoltzTraP code.^46^ The denser *k*-mesh of 24 × 24 × 24 was used for the calculations of the TE properties such as Seebeck coefficient, electrical conductivity, thermal conductivity, specific heat and magnetic susceptibility. The Seebeck coefficient *S* is related to carrier concentration *via* the Mott formula as follows:^47^1
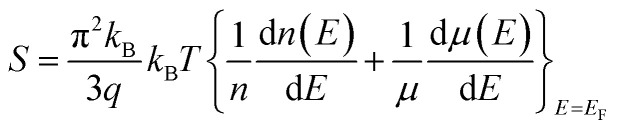
where *E*_F_ is the Fermi energy, *q* is the electron charge, *n* is the carrier concentration and *k*_B_ is Boltzmann constant.

Electrical conductivity is related to carrier concentration as follows:^48^2
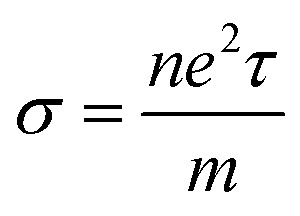
where *m* is the electron mass, *τ* is the relaxation time and *n* is the carrier concentration. Electronic specific heat is related to carrier concentration and chemical potentials as follows:^49^3
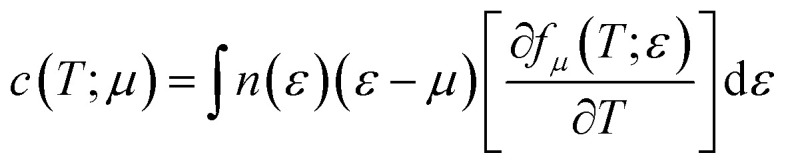


Pauli magnetic susceptibility is related to carrier concentration and chemical potentials as follows:^49^4
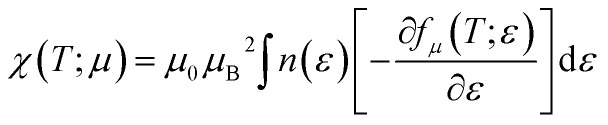
where *μ*_B_ and *μ*_0_ are the Bohr magneton and vacuum permeability, respectively. To obtain the phonon spectrum and the phonon density of states (PhDOS), herein, eight dynamical matrices were calculated using the (4 × 4 × 4) *q*-point mesh. To calculate the optical properties of the compound, random phase approximation (RPA) was used to derive the imaginary part of the dielectric function:^50^5
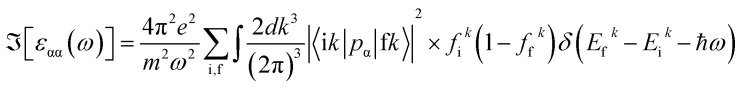
where |i*k*〉 represents the state vector for the initial position and |f*k*〉 represents the state vector for the final position. *f*_i_^*k*^ and *f*_f_^*k*^ represent the Fermi distribution functions of the occupied and unoccupied states, respectively.

The real part of the complex dielectric function was expanded from the imaginary part using the Kramers–Kronig relations as follows:^51–54^6
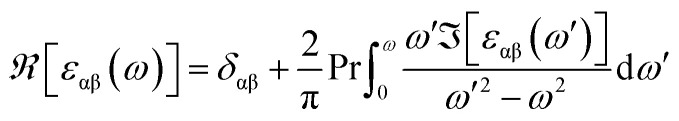
where Pr denotes the Cauchy principal part of the integral. To achieve accurate optical spectra, it is necessary to perform optical calculations with a highly dense first Brillouin zone.^55^ Thus, the 58 × 58 × 58 highly dense *k*-mesh was considered in our optical calculations.

## 3. Results and discussion

### 3.1. Structural properties

**Fig. 1:**
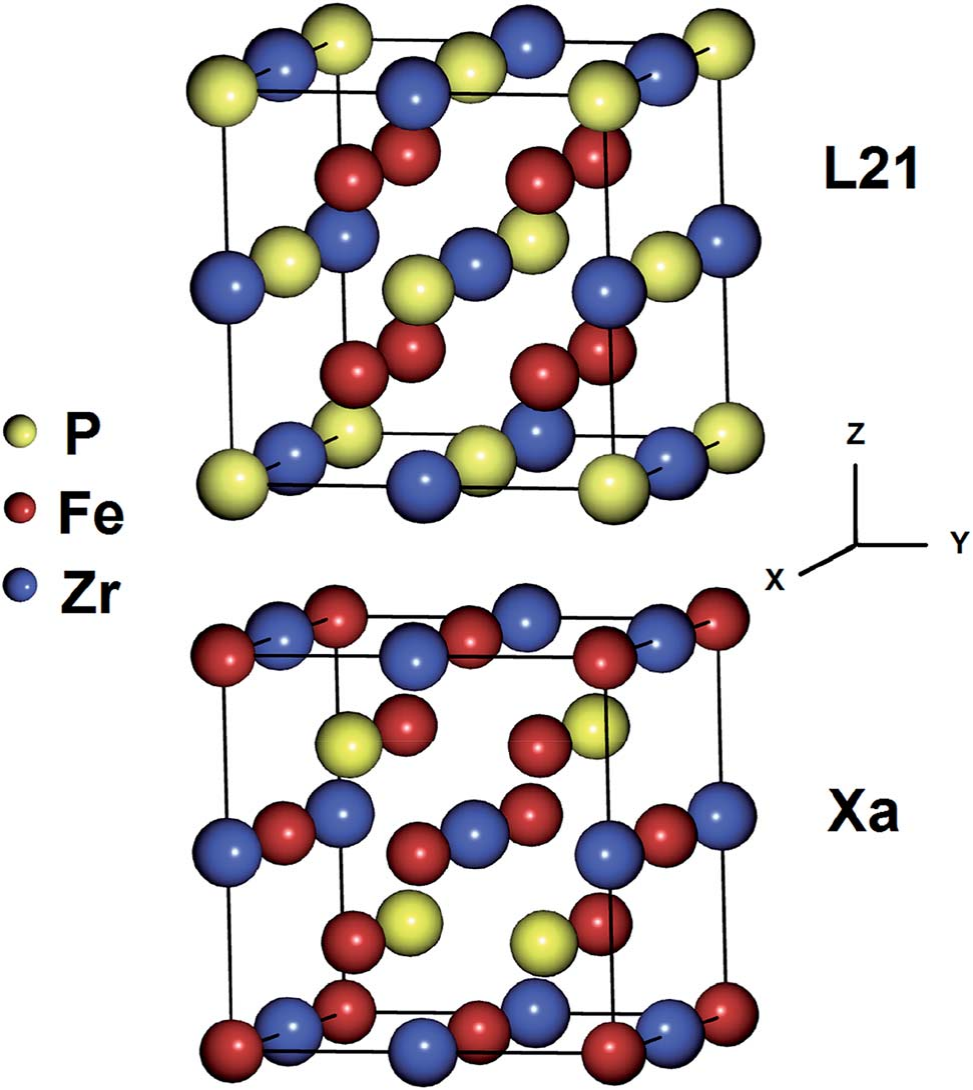
Crystal structures of the Fe_2_ZrP Heusler alloy in L2_1_ and X_a_ prototypes.

The Heusler alloys crystallize in the L2_1_ and X_a_ structures, which belong to the *Fm*3*m* (no. 225) and *F*43*m* (no. 216) space groups, respectively.^56^ The L2_1_ structure is represented by the general formula X_2_YZ, where X and Y are transition metals and Z is a main group element. The X atoms occupy the Wyckoff positions 4a (0, 0, 0) and 4c (1/2, 1/2, 1/2), and the Y and the Z atoms are located at 4b (1/4, 1/4, 1/4) and 4d (3/4, 3/4, 3/4), respectively.^57^ In the X_a_ structure, the X atoms are placed at the two Wyckoff positions (0, 0, 0) and (0.25, 0.25, 0.25), whereas the Y and Z atoms are located at (0.5, 0.5, 0.5) and (0.75, 0.75, 0.75), respectively. The differences between both the abovementioned structures are shown in Fig. 1.

**Fig. 2:**
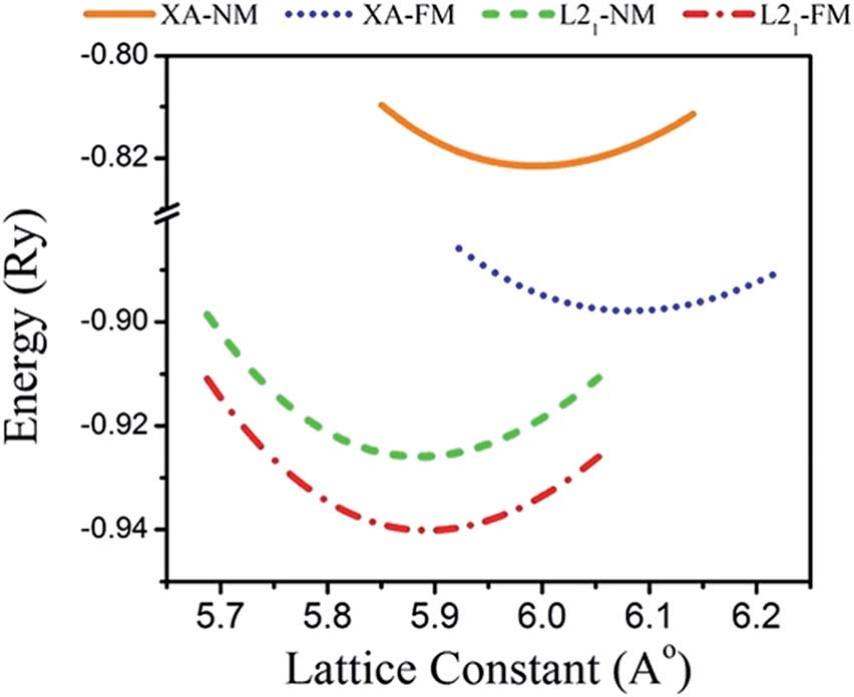
Calculated total energy as a function of the lattice constant of the Fe_2_ZrP compound in L2_1_- and X_a_-type structures.

Total energies *versus* lattice constant were calculated for both the L2_1_ and the X_a_ structures, and the results for the ferromagnetic (FM) and non-magnetic (NM) states are shown in Fig. 2. According to this figure, ferromagnetic order configuration in the L2_1_ structure was found to be the most stable ground state phase as compared to other phases. The calculated values of lattice parameter (*a*_0_), bulk modulus (*B*_0_ in GPa) and the pressure derivative of bulk modulus 
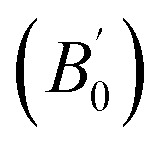
 at the equilibrium lattice constant are presented in Table 1. In the previous study, the total energies were plotted in terms of the lattice volume, and it was concluded that the ferromagnetic order configuration in the L2_1_ structure was the most stable phase; this confirmed the results of the present study.^42^

**Table 1:** The values of the optimized structural parameters

Compound	Prototype	*a*_0_ (Å)	*B*_0_ (GPa)	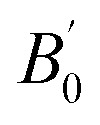
Fe_2_ZrP	L2_1_-FM	5.870	200.74	4.53
	X_a_-FM	6.060	136.64	4.27
	L2_1_-NM	5.860	199.64	4.95
	X_a_-NM	6.960	167.49	5.23

### 3.2. Vibrational properties

**Fig. 3:**
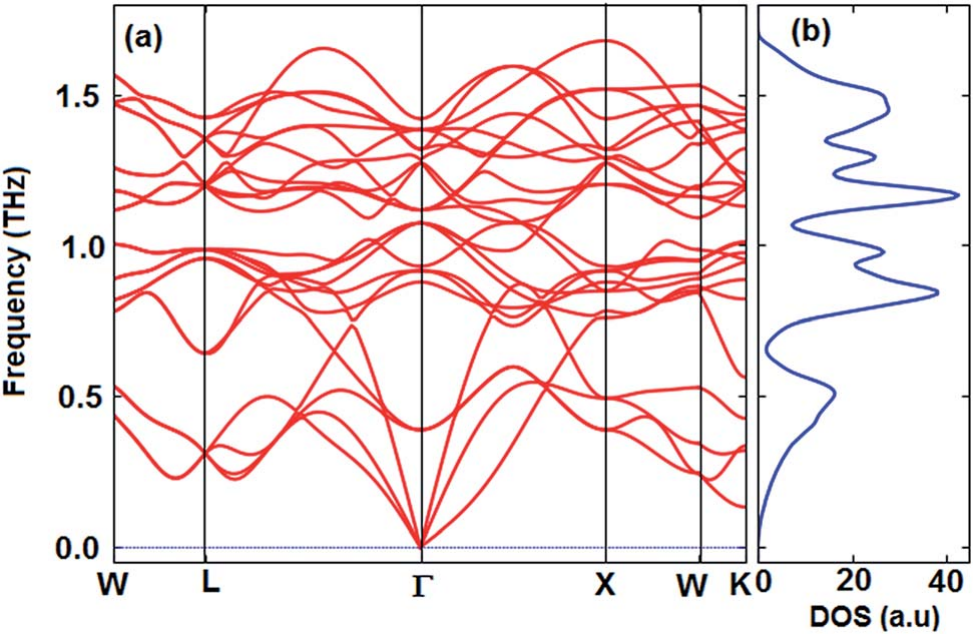
The calculated (a) phonon dispersion and (b) phonon density of states of the Fe_2_ZrP compound.

The thermoelectric properties of materials are often due to the movement of phonons. Materials that have a positive phonon frequency are thermally stable. In previous theoretical studies, to ensure thermoelectric properties, phonon properties were investigated first.^58–62^ The calculated phonon dispersion curve along the W–L–Γ–X–W–K directions and phonon density of states (PhDOS) are shown in Fig. 3. The calculated results show that the Fe_2_ZrP crystal is dynamically stable at zero pressure as no negative frequencies (imaginary modes) exist in the entire Brillouin zone. As observed from Fig. 3a, three vibrational modes below 0.5 THz are acoustic branches, and the remaining vibrational modes are optical modes. The number of optical modes is 3N-3. Therefore, the Fe_2_ZrP crystal with *N* = 4 atoms in the primitive cell exhibits three acoustic and nine optical modes. The acoustic bands are contributed by the Zr element due to its larger atomic mass.^63^ The highest frequency at Γ point is about 1.4 THz. According to Fig. 3b, there is no phonon anomaly in the phonon density of states. The heavier atoms are at low frequencies in the range of 0–0.5 THz.

### 3.3. Electronic band structure and density of states

**Fig. 4:**
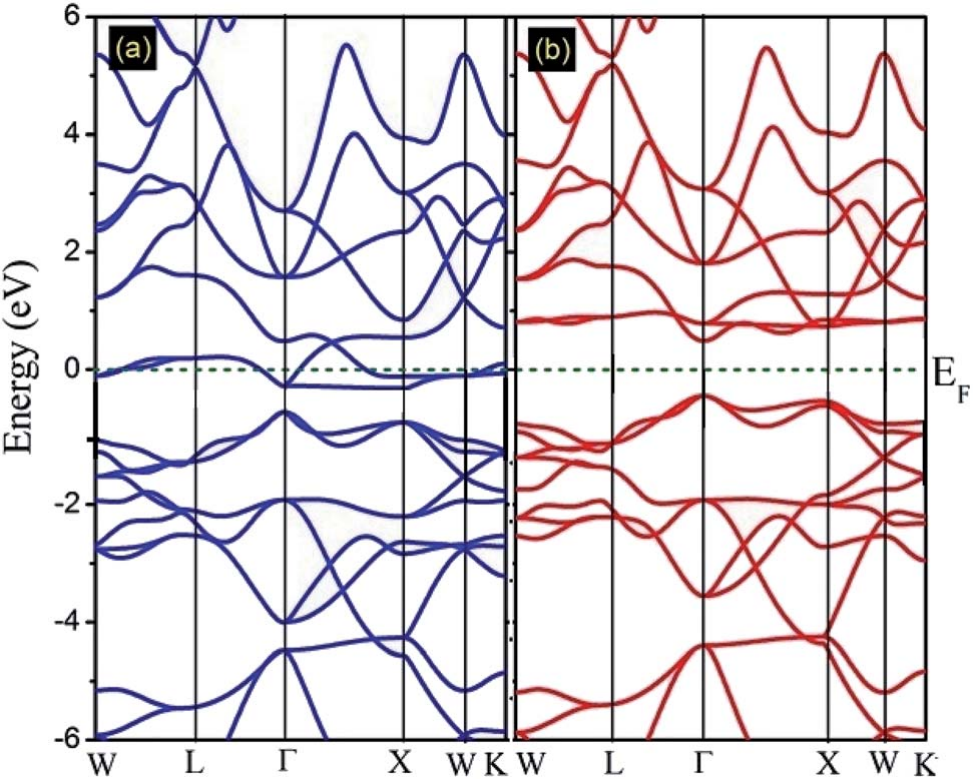
Calculated spin-polarized band structures of the Fe_2_ZrP compound for (a) spin-up and (b) spin-down electrons.

In this subsection, the band structure and density of states of the Fe_2_ZrP compound have been discussed. Fig. 4 illustrates the spin-resolved band structures of the Fe_2_ZrP compound along the higher symmetry direction of the Brillouin zone in the majority (Fig. 4a) and minority (Fig. 4b) spin channels. The zero of the energy scale shows the position of the Fermi level. According to Fig. 4a, the spin-up band structure crosses the Fermi level, clearly showing a strong metallic nature. The Fe_2_ZrP compound exhibits a direct semiconductor behavior in the spin-down channel, with the top of the valence band and the bottom of the conduction band located at Γ_V_ → Γ_C_ (Fig. 4b). We found the direct band gap of about 0.485 eV near the high symmetry direction Γ point. It is obvious that Fe_2_ZrP is metallic and semiconductive in the majority and minority spin channels, respectively. This suggests that the Fe_2_ZrP compound exhibits half-metallic ferromagnetic properties. In addition, Canko *et al.* have shown that the Fermi level is located within the band gap of the spin-down channel but crosses the valence band of the spin-up channel; this is in accordance with the findings of the present study. They found a direct band gap near the high symmetry direction Γ.^42^

**Fig. 5:**
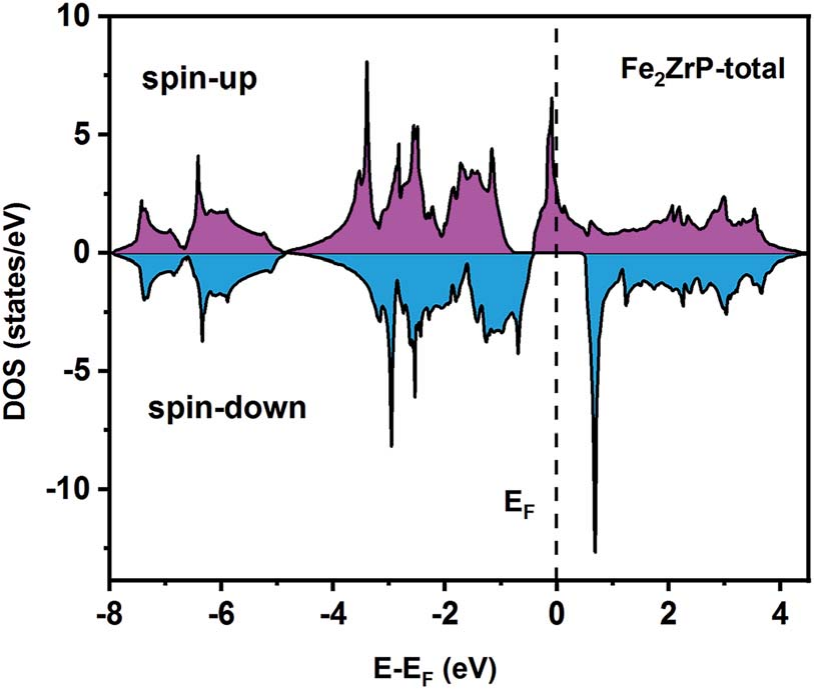
Spin-polarized total density of states of the Fe_2_ZrP compound.

**Fig. 6:**
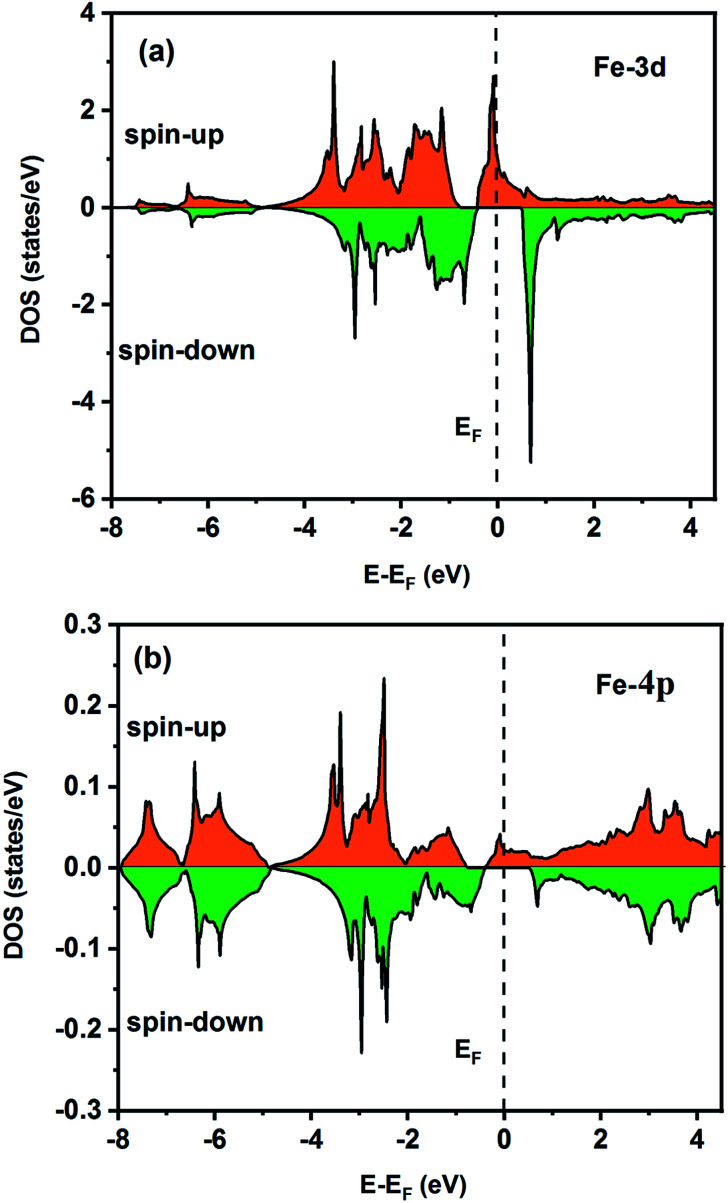
Spin-polarized partial density of states for the Fe atom in the Fe_2_ZrP compound: (a) 3d and (b) 4p orbitals.

**Fig. 7:**
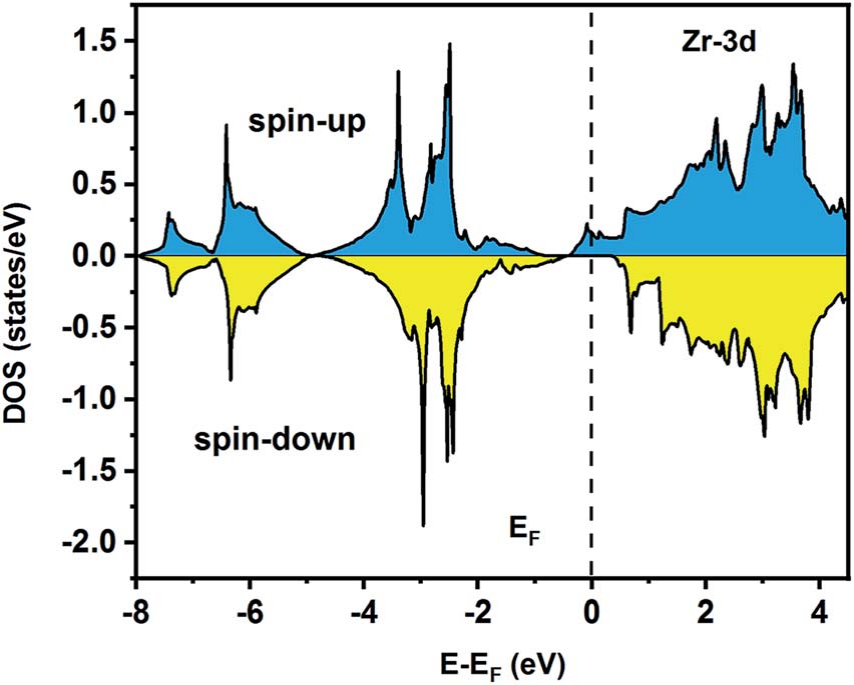
Spin-polarized partial density of states for the Zr atom in the Fe_2_ZrP compound.

**Fig. 8:**
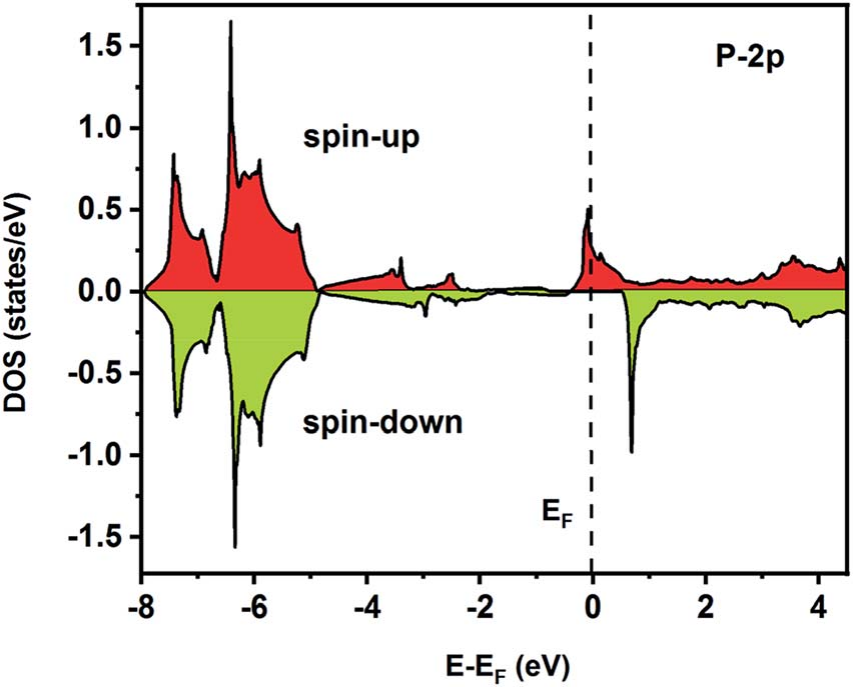
Spin-polarized partial density of states for the P atom in the Fe_2_ZrP compound.

To understand the nature of the electronic states of the Fe_2_ZrP compound at its equilibrium lattice constant, the spin-polarized total density of states (DOS) and partial density of states (PDOS) are displayed in Fig. 5–8. In the previous study on this material, only DOS was examined; on the other hand, in the present theoretical study, in addition to DOS, PDOS was studied in more detail for the better understanding of the electronic structure of this compound.^42^ The energy with respect to the Fermi level is signified by a dashed line. As shown in Fig. 5, in the valence band near the Fermi level, the minority (spin-down) and majority (spin-up) spins are semiconductor and metallic, respectively. This confirms that the compound has a half-metallic behavior. According to Fig. 6a, both spin channels mainly originate from the Fe-3d states, with a small contribution from the Zr and P atoms with s and p orbitals. The Fe-4s, Zr-5p, Zr-4s and P-1s states have a slight effect on the formation of the half-metallic band gap. The transition metal Fe and Zr-3d-states make the main contributions to both spin configurations in the energy range from −4 eV to 4 eV. The electrons at *E*_F_ are fully polarized as the density of spin-up or spin-down channels equals zero. For the Fe_2_ZrP compound, the energy gap located at *E*_F_ leads to a 100% spin polarization.

**Fig. 9:**
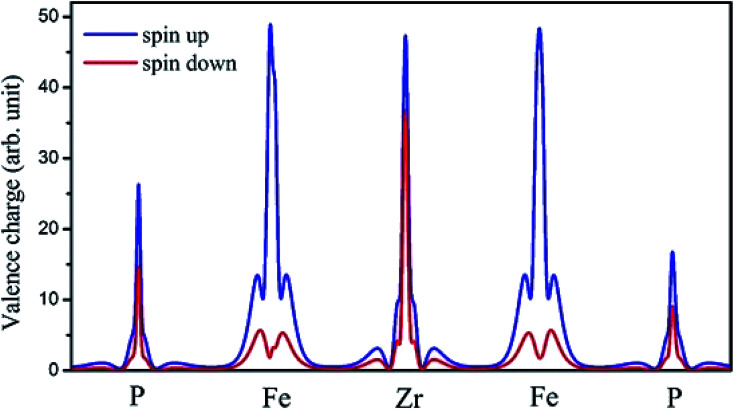
The on-site spin-polarized valence charge density of Fe_2_ZrP elements.

The on-site spin-polarized valence charge density was calculated and is illustrated in Fig. 9 to discuss the origin of magnetic properties in more details. In addition, we selected a crystal direction and made all atoms to lie in this direction to compare charge accumulation between different atoms. As can be observed from Fig. 9, there is an exchange splitting in the valence charge density for all atoms. However, the main difference between spin-up and -down channels is related to the Fe atom. Therefore, similar to other full Heusler alloys X_2_YZ, the main contribution to the magnetic properties is provided by the X (herein, Fe) atoms. The spin-polarized total and atom-projected DOS of the Fe_2_ZrP compound are in agreement with a previous study.^42^

### 3.4. Thermoelectric properties

The TE properties were calculated in the constant relaxation time approximation within the semi-classical Boltzmann theory using the Boltztrap package.^46^ The calculated properties were plotted for three considered temperatures: 300, 600, and 800 K.

**Fig. 10:**
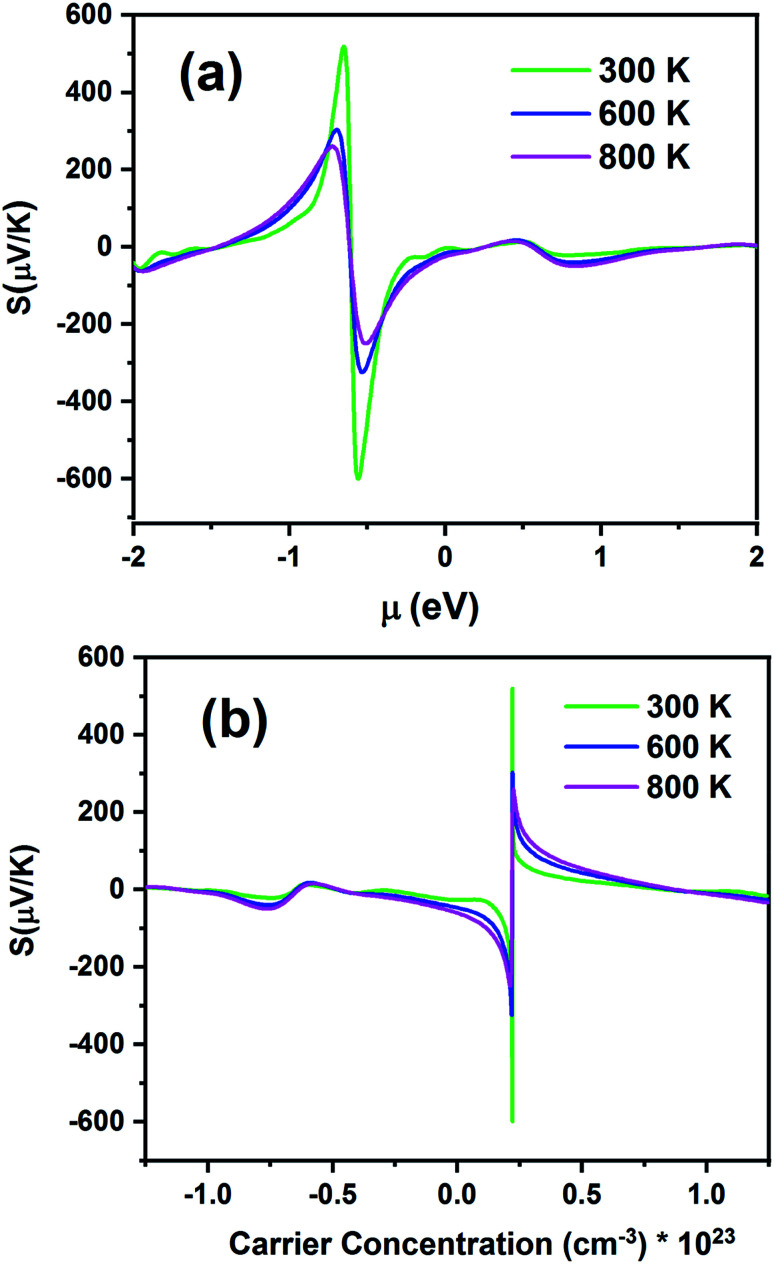
Seebeck coefficients of the Fe_2_ZrP compound as a function of (a) chemical potential and (b) carrier concentration.

Fig. 10 presents the *S* of the Fe_2_ZrP compound as a function of chemical potential (*μ*) in the range from −2 eV to 2 eV (Fig. 10a) and carrier concentration (Fig. 10b). Fig. 10a shows two peaks, which are located at the chemical potentials of −0.65 and −0.55 eV. The Seebeck coefficient inclined rapidly to zero outside this range. As the temperature increased, *S* decreased because of the increase in thermal energy. This indicates that this material has a good thermoelectric performance. The maximum value of *S* is 512 μV K^−1^ at 300 K. For a higher temperature (800 K), *S* is slightly decreased to 260 μV K^−1^. The negative and positive *S* peaks are −606 and 512 μV K^−1^ at 300 K, −324, and 301 μV/K at 600 K and −253 and 260 μV K^−1^ at 800 K, respectively. The positive and negative values of the chemical potential *μ* indicate that the dopants are electrons (n-type) and holes (p-type), respectively. According to Fig. 10b, the maximum value of *S* is obtained for p-type doping, and the optimum carrier concentration is 0.22 × 10^23^ cm^−3^.

According to eqn (1), *S* at different temperatures is higher for smaller concentrations. In Table 2, a comparison between the present theoretical study and previous studies is made.^64–67^ According to this table, as the temperature increases, *S* decreases, and the Fe_2_ZrP compound has suitable values for thermoelectric application. The experimental results of other studies related to the Seebeck coefficient of Heusler compounds are presented in Table 3.^38,39,41^ By comparing the Tables 2 and 3, we concluded that the Fe_2_ZrP compound had good potential for experimental production.

**Table 2:** A comparison between the Seebeck coefficients of the present and other studies. S measurement unit is μV K^−1^

Temperature (K)	Fe_2_ZrP	TiZrNiSn^64^	TiSiSb^65^	Zr_2_MnAl^66^	Sc_2_FeSi^67^
300	500	300	200	700	400
600	300	240	150	500	300
800	270	190	100	470	200

**Table 3:** The Seebeck coefficients obtained in other experimental studies (Heusler compounds) at 800 K. S measurement unit is μV K^−1^

Zr_0.8_Hf_0.2_CoSb_0.9_Sn_0.1_ (ref. 38)	240
Zr_0.6_Hf_0.4_CoSb_0.9_Sn_0.1_ (ref. 38)	225
Zr_0.5_Hf_0.5_CoSb_0.8_Sn_0.2_ (ref. 39)	220
Hf_0.3_Zr_0.7_CoSb_0.7_ (ref. 41)	170

**Fig. 11:**
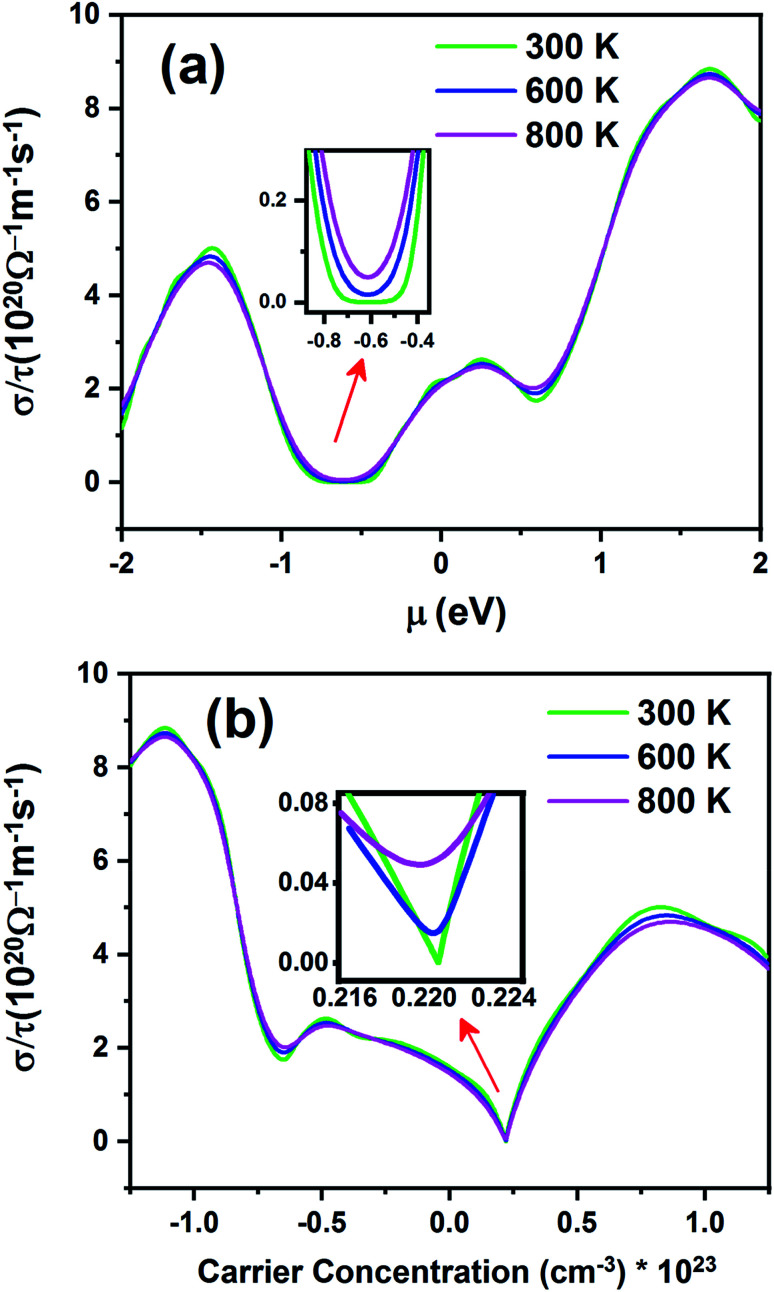
The electrical conductivity of the Fe_2_ZrP compound as a function of (a) chemical potential and (b) carrier concentration.

Fig. 11 shows electrical conductivity (*σ*/*τ*) as a function of chemical potential (Fig. 11a) and carrier concentration (Fig. 11b) at different temperatures. Unlike the Seebeck coefficient, the electrical conductivity displays similar behavior at all temperatures.

According to Fig. 11a, the electrical conductivity increases with an increase in chemical potential. With an increase in chemical potential, the carrier concentration increases, and an increase in mobility increases the conductivity. The inset image in Fig. 11a shows that electrical conductivity is zero in the range from −0.49 to −0.73 at 300 K. As shown in Fig. 11b, the n-type doped compound has higher electrical conductivity than the p-type doped compound. According to eqn (2), the electrical conductivity increases with an increase in carrier concentration. The inset image in Fig. 11b shows that *σ*/*τ* is zero at 300 K in the p-type doping area, where the carrier concentration is about 0.22 × 10^23^ cm^−3^.

**Fig. 12:**
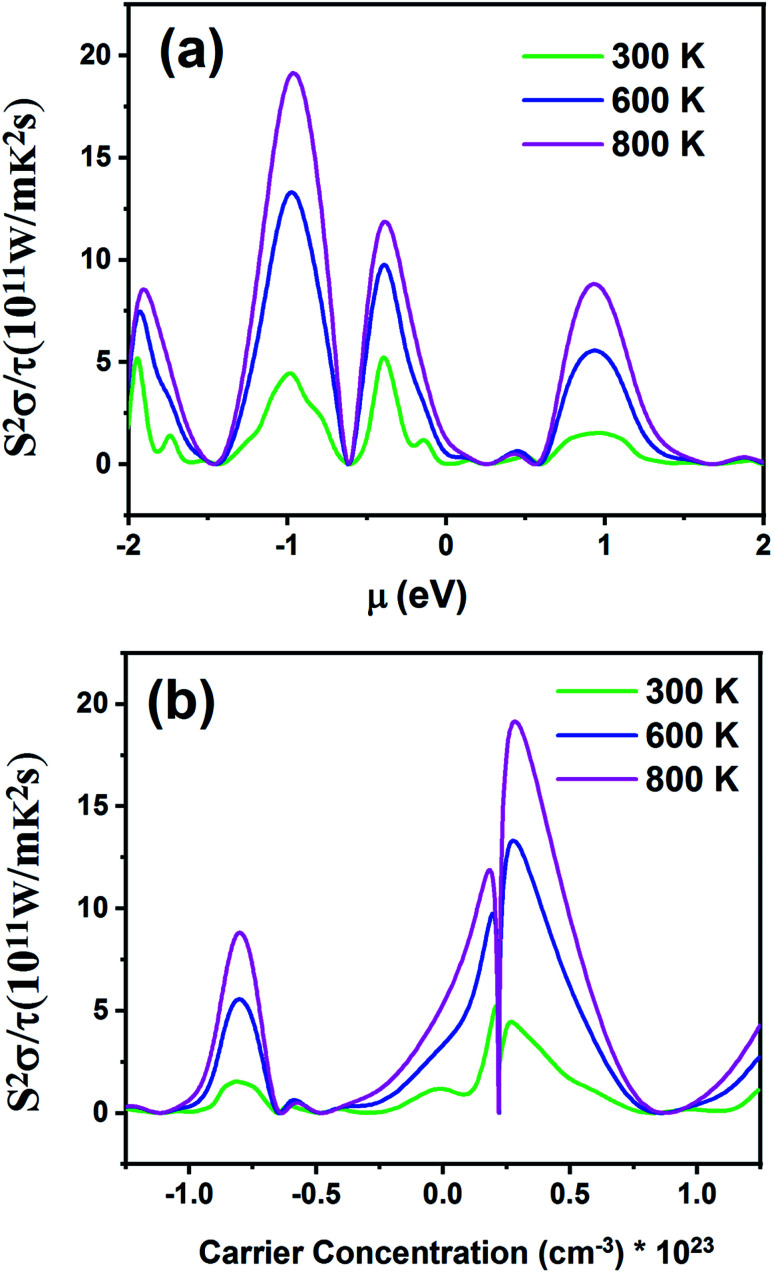
The power factor of the Fe_2_ZrP material as a function of (a) chemical potential and (b) carrier concentration.

Fig. 12 displays the electronic power factor values (*S*^2^*σ*) as a function of chemical potential relative to the Fermi level (Fig. 12a) and carrier concentration (Fig. 12b) at different temperatures. This quantity investigates the efficiency of the thermoelectric materials. According to Fig. 12a, as the temperature increases, power factor also increases. The maximum value of power factor is 19.21 × 10^11^ W m^−1^ K^−2^ s^−1^ for negative chemical potential at 800 K. At room temperature, the power factor is slightly decreased to 4.43 × 10^11^ W m^−1^ K^−2^ s^−1^. As shown in Fig. 12b, the maximum value of power factor is located in the p-type doping area (0.22 × 10^23^ cm^−3^), which is higher than that of the n-type doping area (8.61 × 10^11^ W m^−1^ K^−2^ s^−1^).

**Fig. 13:**
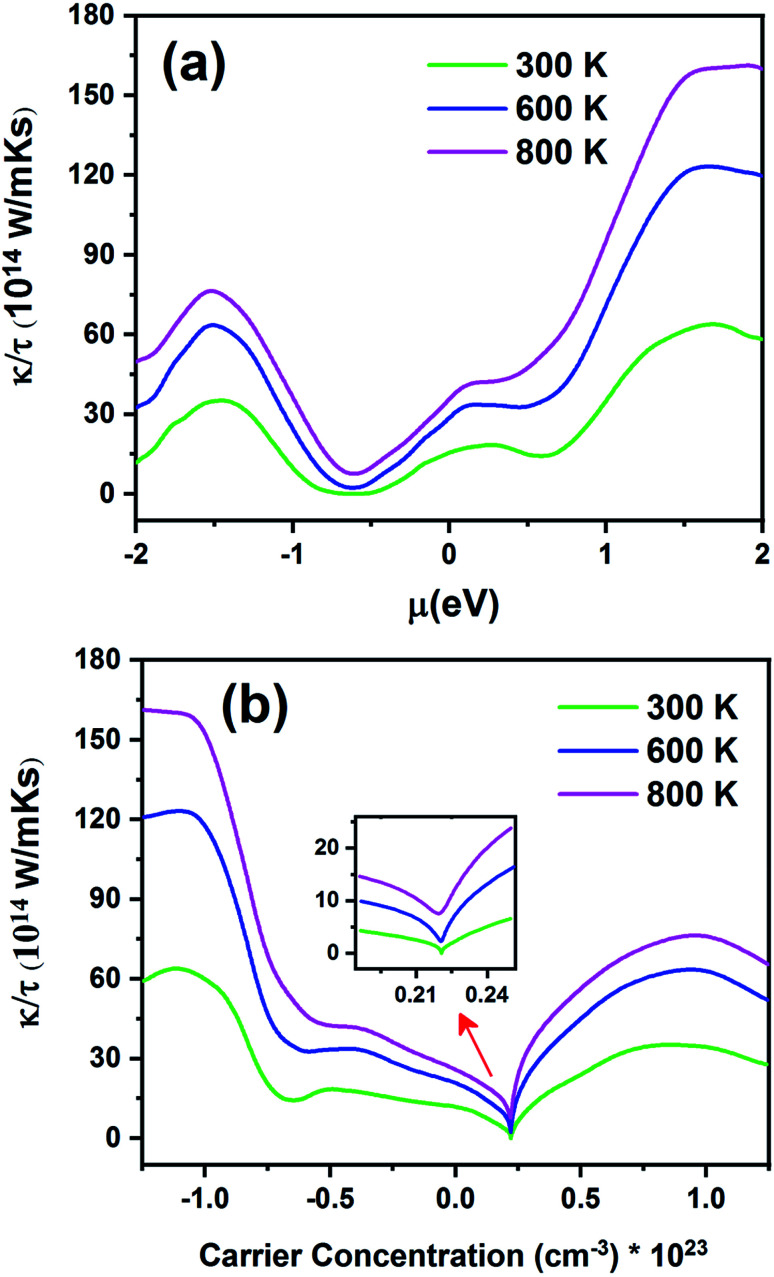
The electronic thermal conductivity of the Fe_2_ZrP compound as a function of (a) chemical potential and (b) carrier concentration.

Fig. 13 displays the electronic thermal conductivity (*κ*/*τ*) as a function of chemical potential (Fig. 13a) and carrier concentration (Fig. 13b) at three constant temperatures (300, 600 and 800 K). According to this figure, as the temperature increases, thermal conductivity also increases. To increase the thermoelectric properties, the materials must have large *S*, high electrical conductivity, and low thermal conductivity.^68^ Therefore, the optimum temperature to obtain lower *κe*/*τ* is 300 K. According to Fig. 13a, the thermal conductivity increases with an increase in chemical potential. The thermal conductivity is zero in the range from −0.68 to −0.55 at 300 K. As shown in Fig. 13b, the n-type doped compound has higher thermal conductivity than the p-type doped compound. Moreover, the electrical conductivity increases with an increase in carrier concentration. The inset image in Fig. 13b shows that *κ*/*τ* is zero at 300 K in the p-type doping area where the carrier concentration is about 0.22 × 10^23^ cm^−3^.

**Fig. 14:**
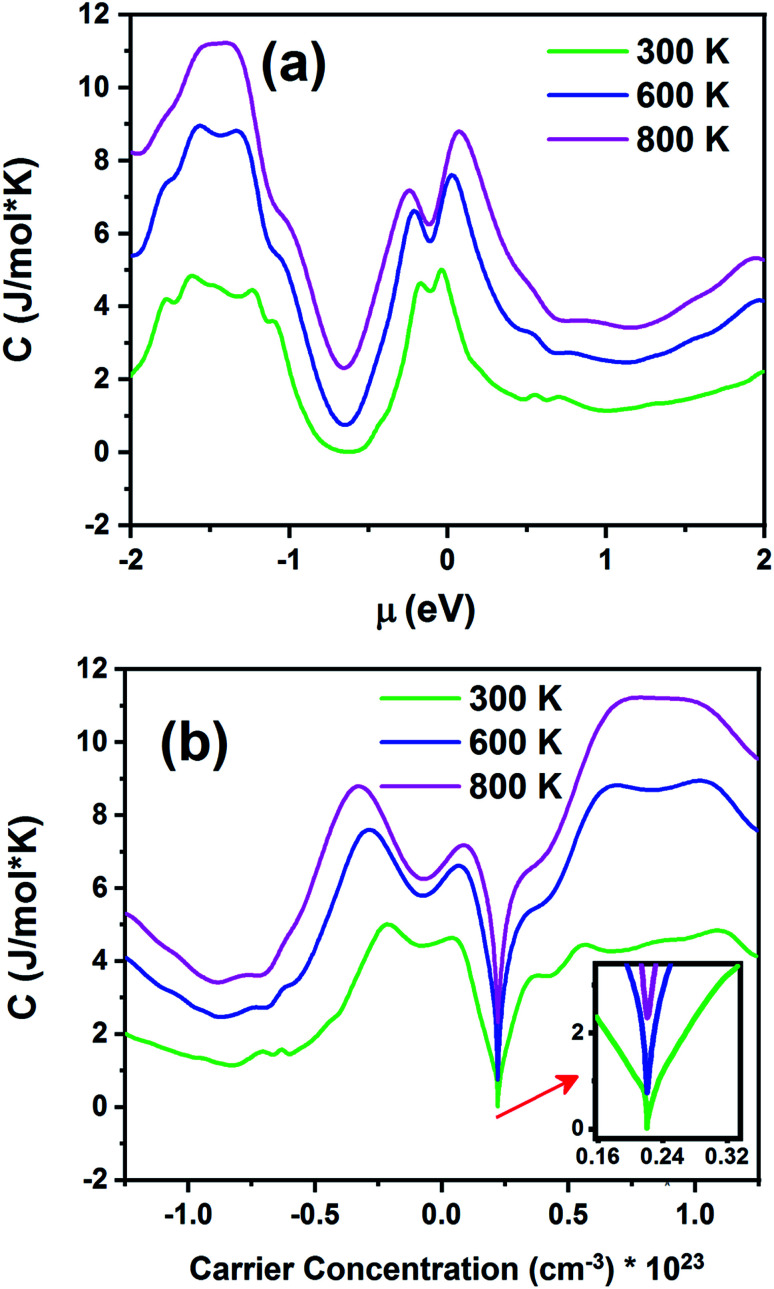
The electronic specific heat of the Fe_2_ZrP material as a function of (a) chemical potential and (b) carrier concentration.

Fig. 14 displays the electronic specific heat (*c*) as a function of chemical potential (Fig. 14a) and carrier concentration (Fig. 14b) at different temperatures. According to this figure, as the temperature increases, specific heat also increases. According to eqn (3), the specific heat increases with an increase in carrier concentration and chemical potential. According to Fig. 14a, the maximum value of the specific heat is 11.22 J (mol K)^−1^ for negative chemical potential at 800 K. At room temperature, the specific heat is decreased to 4.81 J mol^−1^ K^−1^. As shown in Fig. 14b, the maximum value of specific heat is obtained in the p-type doping area (11.19 × 10^23^ cm^−3^), which is higher than that of the n-type doping area (8.7 J mol^−1^ K^−1^). The inset image in Fig. 14b shows that the specific heat is zero at 300 K in the p-type doping area where the carrier concentration is about 0.22 × 10^23^ cm^−3^.

**Fig. 15:**
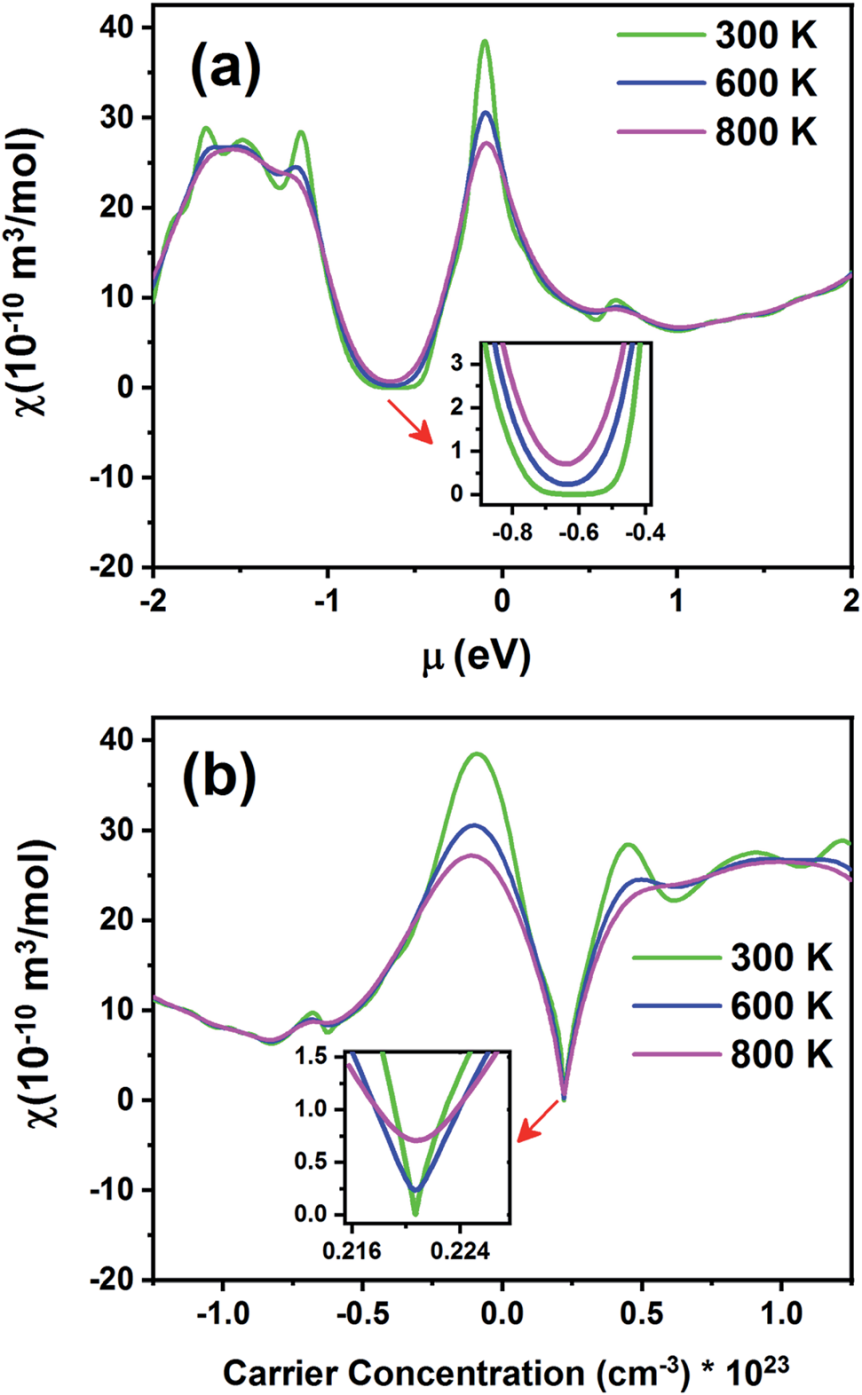
The Pauli magnetic susceptibility of the Fe_2_ZrP material as a function of (a) chemical potential and (b) carrier concentration.

Fig. 15 exhibits the Pauli magnetic susceptibility (*χ*) as a function of chemical potential (Fig. 15a) and carrier concentration (Fig. 15b) at different temperatures. According to Fig. 15a, the Pauli magnetic susceptibility displays an almost similar behavior at all temperatures except near the Fermi level. At this point, as the temperature increases, *χ* decreases. The maximum value of *χ* is 38.65 × 10^−10^ m^3^ mol^−1^ at 300 K. For a higher temperature (800 K), *χ* is slightly decreased to 27.12 × 10^−10^ m^3^ mol^−1^. As observed from the inset image shown in Fig. 15a, the Pauli magnetic susceptibility is zero in the range from −0.68 to −0.55 at 300 K. According to eqn (4) and Fig. 15b, the Pauli magnetic susceptibility increases with an increase in carrier concentration. As shown in Fig. 15b, the maximum value of specific heat is obtained in the n-type doping area (−0.08 × 10^23^ cm^−3^). The inset image in Fig. 15b shows that the Pauli magnetic susceptibility is zero at 300 K in the p-type doping area where the carrier concentration is about 0.22 × 10^23^ cm^−3^.

**Fig. 16:**
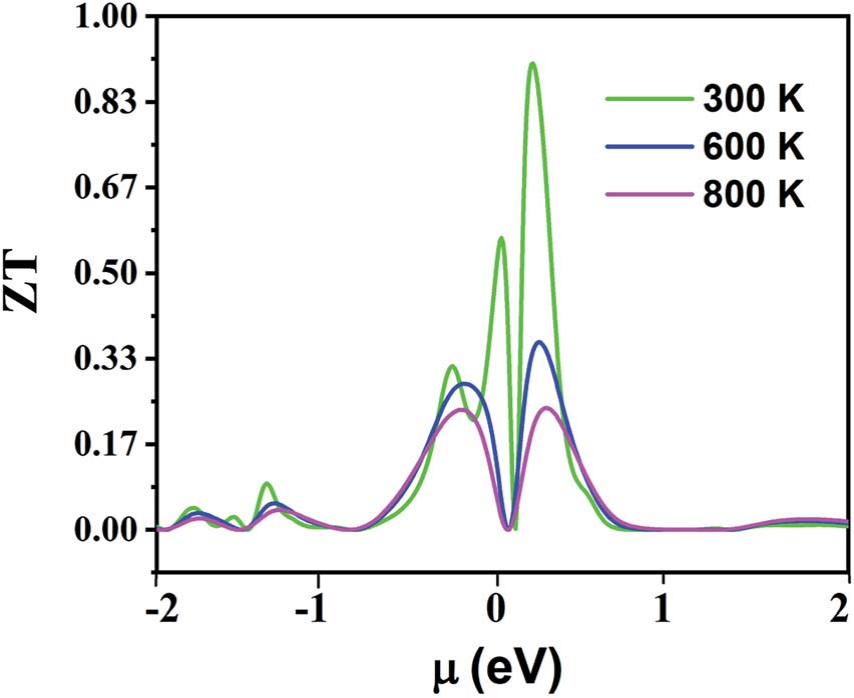
The electronic figure of merit of Fe_2_ZrP as a function of chemical potential.

Fig. 16 displays the electronic figure of merit (*ZT*) values of the Fe_2_ZrP compound as a function of chemical potential at three constant temperatures (300, 600 and 800 K). This quantity investigates the efficiency of the thermoelectric materials. According to this figure, as the temperature increases, *ZT* decreases. At all temperatures, *ZT* is low where chemical potential is negative. According to this figure, the best temperature for thermoelectric applications is 300 K because *ZT* has a good value in the negative and positive fields of chemical potential. In Table 4, a comparison between the present theoretical study and previous studies is shown.^64–66,69^ According to the table, the Fe_2_ZrP compounds are good thermoelectric materials. The experimental results of other studies on Heusler compounds based on the figure of merit are also presented in Table 5.^38,39,41^ By comparing the Tables 4 and 5, we conclude that the Fe_2_ZrP compounds have a suitable figure of merit.

**Table 4:** A comparison between the *ZT* of the present and other studies (in the positive chemical potential)

Temperature K	Fe_2_ZrP	GdMnO_3_ (ref. 69)	TiZrNiSn^64^	TiSiSb^65^	Zr_2_MnAl^66^
300	0.89	0.68	0.40	0.38	0.87
600	0.33	0.34	0.65	0.29	0.65
800	0.21	0.4	0.60	0.24	0.45

**Table 5:** The *ZT* of other experimental works (Heusler compounds) at 600 K

Zr_0.8_Hf_0.2_CoSb_0.9_Sn_0.1_ (ref. 38)	0.45
Zr_0.6_Hf_0.4_CoSb_0.9_Sn_0.1_ (ref. 38)	0.52
Zr_0.5_Hf_0.5_CoSb_0.8_Sn_0.2_ (ref. 39)	0.50
Hf_0.3_Zr_0.7_CoSb_0.7_ (ref. 41)	0.39

### 3.5. Optical properties

Herein, the optical properties of the Fe_2_ZrP compound have been studied using a random phase approximation (RPA) method. To investigate the optical properties of a half-metallic material, it was necessary to consider both intraband and interband contributions in our calculations; due to their transitional nature, the intraband transitions affected only the infra-red and visible ranges of light in the optical spectra.^70^

**Fig. 17:**
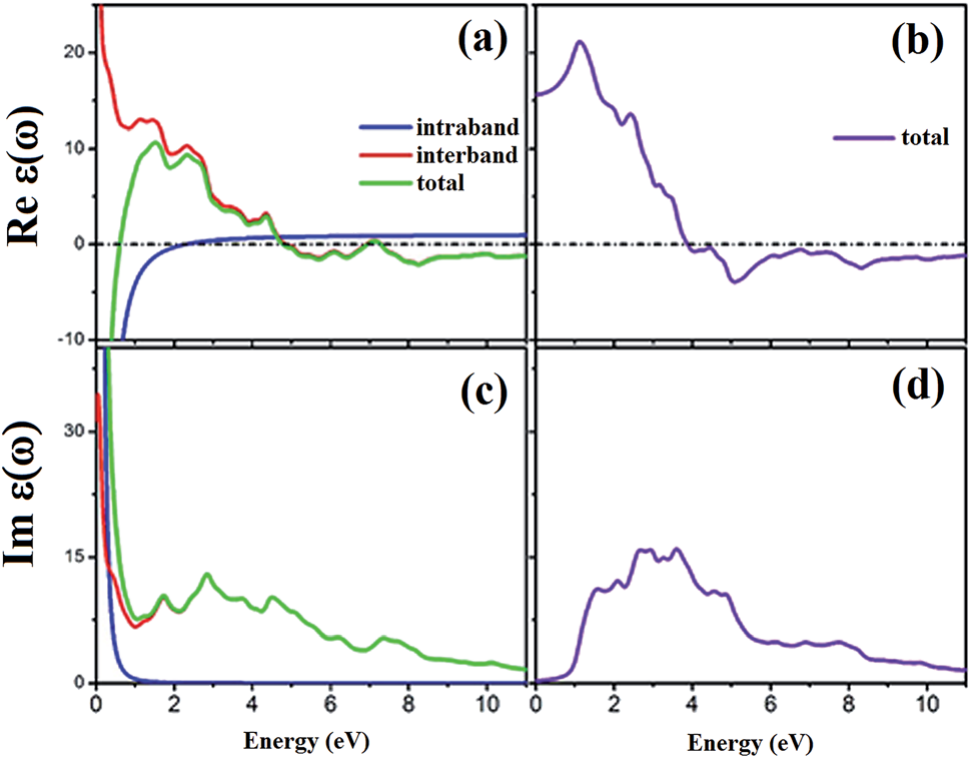
Calculated spin-dependent real part of dielectric function for (a) spin-up and (b) spin-down channels and the imaginary part for (c) spin-up and (d) spin-down channels.

The spin-dependent imaginary and real parts of the dielectric function are illustrated in Fig. 17. The electronic band structure exhibits that the spin-up channel has a metallic behavior, whereas the spin-down channel has a semiconductive behavior. Therefore, intraband transitions occur only for the free electrons of the spin-up channel. As a result, the intraband, interband and total contributions have been plotted only for the spin-up channel. As can be observed, the intraband transitions have the main role in the range of 0–2 eV in the real and imaginary parts of the spin-up channel. This trend refers to the free electron effect in the spin-up channel. For metallic materials in low frequency range, the refractive index *n*(*ω*) is lower than the extinction coefficient *k*(*ω*); thus, the real part of dielectric function has a negative value, *ε*_1_ = *n*^2^ − *k*^2^ < 0. The imaginary part of the dielectric function refers to optical absorption from the occupied states to the unoccupied states. In Fig. 17c, we can see a high value of absorption from zero energy to 2 eV (free electron absorption), whereas there is an absorption threshold in the spin-down imaginary part spectrum (Fig. 17d) that is according to the half-metallic band gap structure of the spin-down channel.

**Fig. 18:**
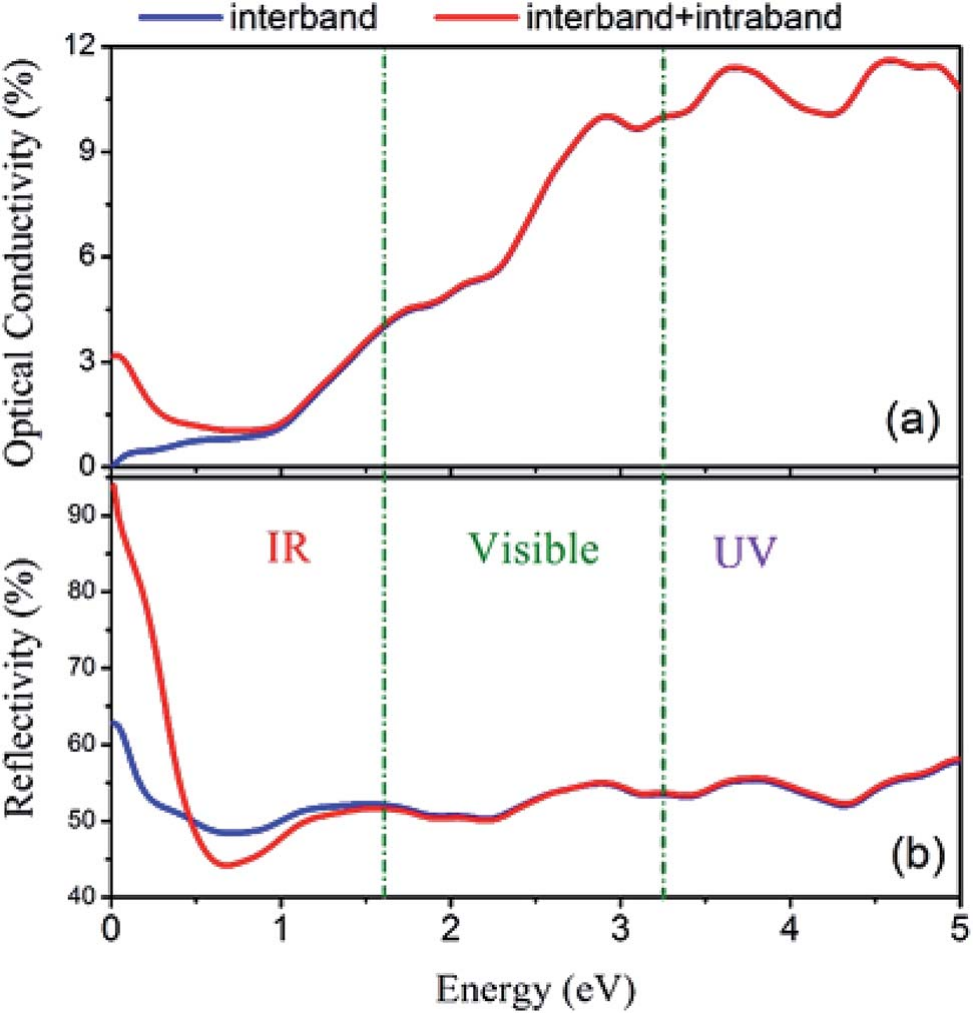
(a) Optical conductivity and (b) reflectivity spectra with and without considering intraband transitions.

Furthermore, the total spectra, *i.e.* spin up plus spin down spectra, of optical conductivity and reflectivity were calculated and are plotted in Fig. 18 with and without intraband transitions. The results indicate that due to the partially occupied states in the spin-up band structure of Fe_2_ZrP around the Fermi level, the intraband contribution has the main role in the infra-red range of optical spectra. This phenomenon leads to a high reflectivity spectrum in the infra-red range of incident light.

## 4. Conclusion

Herein, the electronic, phononic and thermoelectric properties of the Fe_2_ZrP compound were calculated using the DFT and Boltzmann transport theory calculations. It was found that this material was half-metallic with the indirect band gap of 0.485 eV along the Γ_V_–Γ_C_ symmetry line. The phonon density of states and phonon dispersion curves confirm that the Fe_2_ZrP compound is dynamically stable. The results of Boltzmann calculations showed that the Fe_2_ZrP compound exhibited better thermoelectric properties after p-type doping than after n-type doping; the highest *S* value was obtained at the temperature of 300 K upon p-type doping. The thermoelectric and phononic properties of the Fe_2_ZrP compound were considered for the first time in this study. The maximum value of the power factor reaches 19.21 × 10^11^ W m^−1^ K^−2^ at the hole concentration of 0.22 × 10^23^ cm^−3^ and about 8.61 × 10^11^ W m^−1^ K^−2^ s^−1^ at the electron concentration at 800 K. The electrical and thermal conductivity increase with the increasing chemical potential. This study shows that the Fe_2_ZrP compound has a good potential for application in the thermoelectric field. The optical calculations confirm that the intraband contribution has the main role in the low energy ranges (infra-red and visible) of optical spectra.

## Conflicts of interest

There are no conflicts to declare.

## Supplementary Material
